# Efficacy of intravenous acetaminophen in multimodal management for pain relief following total knee arthroplasty: a meta-analysis

**DOI:** 10.1186/s13018-018-0950-7

**Published:** 2018-10-11

**Authors:** Song-bo Shi, Xing-bo Wang, Jian-min Song, Shi-fang Guo, Zhi-xin Chen, Yin Wang

**Affiliations:** Orthopaedics Department, Gansu Provincial Hospital, Lanzhou, 730000 Gansu China

**Keywords:** Acetaminophen, Pain control, Total knee arthroplasty, Meta-analysis

## Abstract

**Background:**

The efficacy of intravenous acetaminophen in multimodal pain management in patients undergoing total knee arthroplasty (TKA) is controversial. The purpose of this meta-analysis was to compare the efficacy of intravenous acetaminophen versus placebo in TKA.

**Methods:**

Randomized controlled trials (RCTs) or retrospective cohort studies (RCSs) concerning related topics were retrieved from PubMed (1996–June 2018), Embase (1980–June 2018), and the Cochrane Library (CENTRAL June 2018). Any studies comparing intravenous acetaminophen with a placebo were included in this meta-analysis. Meta-analysis results were collected and analyzed by Stata 12.0. Subgroup analysis was performed according to the general characteristics of the patients.

**Results:**

In total, the patients from six studies met the inclusion criteria. Our meta-analysis results indicated that compared with a control group, intravenous acetaminophen was associated with reductions in total morphine consumption and visual analogue scale (VAS) score at postoperative day (POD) 3. However, there was no significant difference in morphine consumption at POD 1 or in VAS at POD 1 or POD 2. Moreover, there was no significant difference in the length of hospital stay.

**Conclusions:**

Based on our results, intravenous acetaminophen in multimodal management has shown better efficacy in pain relief at POD 3 and has morphine-sparing effects. High-quality studies with more patients are needed in the future.

**Electronic supplementary material:**

The online version of this article (10.1186/s13018-018-0950-7) contains supplementary material, which is available to authorized users.

## Background

Total knee arthroplasty (TKA) is being widely used for end-stage osteoarthritis (OA) or rheumatoid arthritis (RA) [1]. However, over 80% of TKA patients experience severe to moderate postoperative pain [2]. Inadequate pain management may result in dissatisfaction, complications, stunted postoperative functional recovery, and longer hospital stays [3, 4]. Conventionally, multimodal pain management is widely recommended and accepted [5, 6]. Multimodal pain management usually includes two or more analgesics, such as opioids, nonsteroidal anti-inflammatory medications, steroid hormones, and epinephrine [7]. It has been reported that rebounding pain in patients treated with multimodal pain management after 24 h postoperatively remains a real problem for surgeons [8, 9]. Furthermore, the use of opioids is frequently associated with some side effects, including gastrointestinal symptoms, autonomic nervous system symptoms, and central nervous system symptoms, among others [10, 11]. Thus, adjunctive pain management medication is needed.

Recently, combination therapy with intravenous (IV) acetaminophen has been used to reduce postoperative pain, and opioid use across a variety of surgical procedures has also been applied to the TKA [12–14]. Kelly et al. [15] drew the conclusion that IV acetaminophen did not significantly decrease postoperative opioid use in patients who underwent surgical knee procedures. Studies conducted by Blank et al. [16] and Nwagbologu et al. [17] presented similar conclusions. However, O’Neal et al. [18] reported that neither IV nor oral acetaminophen provided better analgesia in patients undergoing TKA. Similar results were reported by subsequent studies [19]. Thus, conclusions concerning the use of IV acetaminophen in reducing postoperative pain and opioid consumption have been inconsistent. Several studies have reported that IV acetaminophen has a beneficial role in reducing pain intensity and morphine consumption after TKA [19, 20]. However, some other studies suggest that the use of acetaminophen in multimodal pain management does not result in improved safety or reduced opioid utilization in hip or knee arthroplasty [17, 21].

Therefore, it is necessary to investigate whether IV acetaminophen as an adjunctive pain management medication provides better analgesic effects, as well as whether it reduces opioid consumption in patients after TKA. The purpose of the current meta-analysis was to compare results concerning the efficacy of IV acetaminophen for pain control in patients undergoing TKA.

## Methods

### Search strategy

**Fig. 1:**
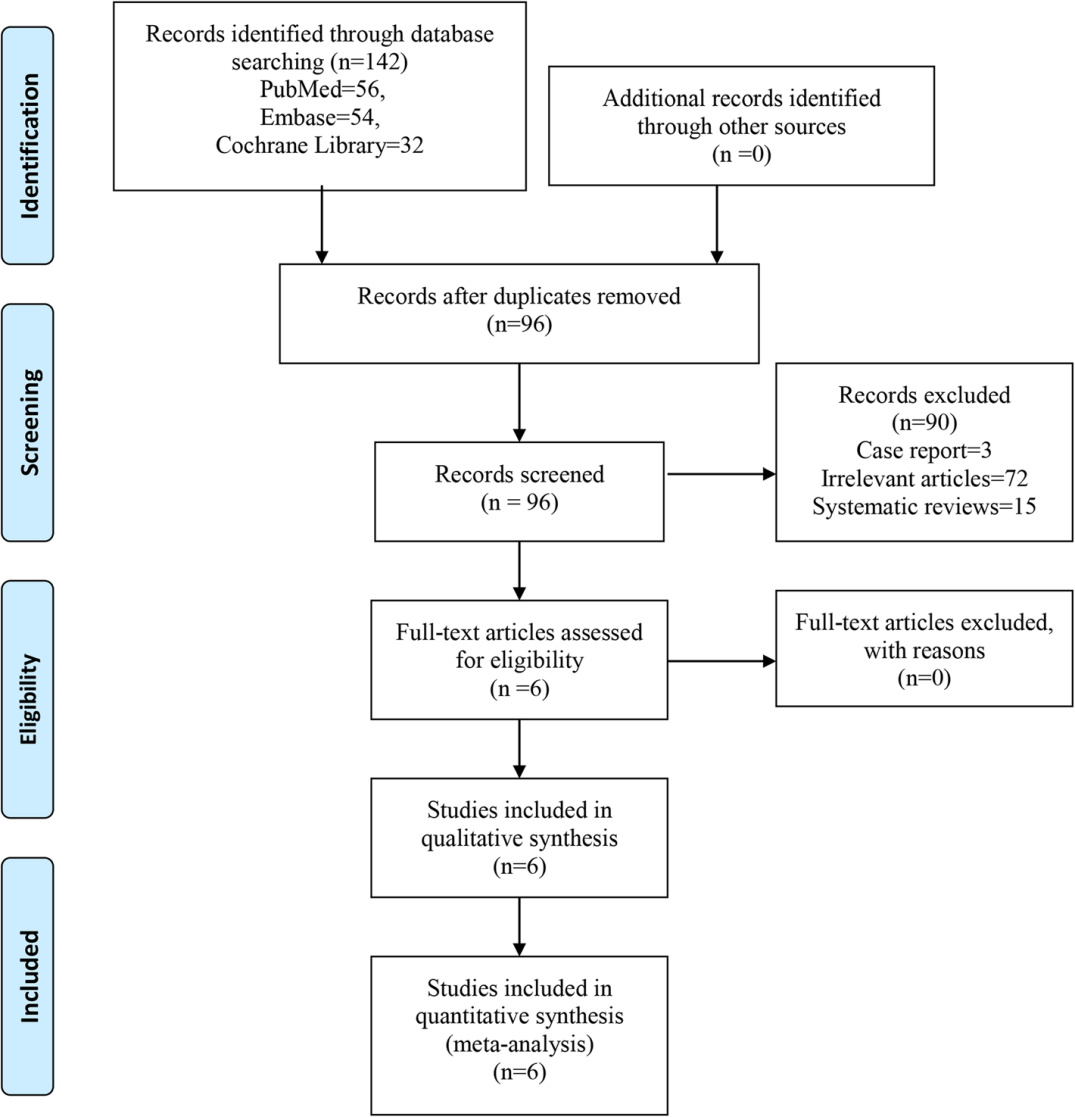
Flow of trials through the meta-analysis

We manually searched randomized controlled trials (RCTs), retrospective cohort studies (RCSs), and cohort studies through PubMed (1996–June 2018), Embase (1980–June 2018), and the Cochrane Library (CENTRAL, June 2018). We also searched trials from related references and reviews. The key words and MeSH terms were “total knee arthroplasty,” “total knee replacement,” “TKA,” “TKR,” “Arthroplasty, Replacement, Knee” [MeSH], and “acetaminophen.” These key words or MeSH terms were combined using the Boolean operators “AND” or “OR.” The search results are presented in Fig. 1.

### Inclusion criteria

Studies were included in our meta-analysis provided that they satisfied the condition of meeting the PICOS (patients, intervention, comparator, outcomes, and study design) study quality assurance guidelines. Other inclusion criteria included the following: (1) Patients had undergone TKA. (2) The intervention was intravenous acetaminophen. (3) The comparator was non-intravenous administration of acetaminophen or placebo. (4) The outcomes included morphine equivalent consumption at POD 1, total morphine equivalent consumption, VAS score at 24, 48, and 72 h and length of hospital stay.

### Data extraction

Two reviewers extracted available data from the included studies independently. Extracted data included first author, publication data, participants, age, gender, body mass index, and study design. The primary outcome of our meta-analysis consisted of morphine equivalent consumption at POD 1, total morphine consumption, and VAS score at 24, 48, and 72 h postoperatively. Secondary outcomes consisted of length of hospital stay. We tried emailing the corresponding authors of the studies that used graphical data or had incomplete data. Any disagreement between the two reviewers was resolved by a third reviewer.

### Quality assessment

Quality assessment for RCTs was performed according to the Cochrane Handbook for Systematic Reviews of Interventions. Two authors independently evaluated the risk of bias of the included RCTs based on the following items: random sequence generation, allocation concealment, blinding, incomplete outcome data, selective reporting, and other sources of bias. For non-RCTs, we used the Newcastle-Ottawa scale to evaluate the risk of bias [22]. We considered a study to be of high quality for non-RCTs when a study achieved a score on the Newcastle-Ottawa scale of more than six points.

### Statistical analysis

Stata 12.0 was applied for our meta-analysis. For continuous outcomes, mean difference (MD) with a 95% confidence interval (CI) was used to weigh the effect intervals. We judged the statistical heterogeneity by the *P* value derived using the standard chi-square test. Values of *I*^2^ > 50% were thought to have significant heterogeneity of the outcomes, and a random-effect model was applied for assessment; for others, such as for extracted data, a fixed-effect model was used. We performed subgroup analysis by omitting studies in turn. Subgroup analysis was done according to the study type, anesthesia, allocation concealment, and dose of acetaminophen.

## Results

### Search results and general characteristics

A total of 142 relevant studies were identified by our search strategies. After duplicates were removed, there were 96 studies left to review. After reading the title and abstract, 90 studies were excluded. Finally, 6 studies [15, 17–19, 21, 23] were included in our meta-analysis after full-text reading. Among them, there were 2 RCTs [18, 19] and 4 RCSs [15, 17, 21, 23]. General characteristics of the included RCTs can be seen in Table 1. All of the studies were published in 2014. The ages of the TKA patients ranged from 61 to 75.3 years. Four studies [15, 17–19] administered acetaminophen at a dose of 1000 mg/day, and one study administered acetaminophen at a dose of 4000 mg/day [21].

**Table 1:** General characteristic of the included studies

Author	Country	Age (year)	Study	Dose of acetaminophen	Control	Follow-up	Anesthesia
Kelly [15]	America	63.9/65.3	RCS	1000 mg/day	Placebo	NS	NS
Nwagbologu [17]	Mexico	61/63.9	RCS	1000 mg/day	Placebo	3 days	NS
O’Neal [18]	America	68/70	RCT	1000 mg/day	Placebo	NS	SA
Murata-Ooiwa [19]	Japan	73.6/75.3	RCT	1000 mg/day	Placebo	3 days	SA
Ciummo [23]	America	NS	RCS	NS	Placebo	NS	NS
Huang [21]	America	71.3/71.6	RCS	4000 mg/day	Placebo	3 days	SA/GA

*RCT* randomized controlled trials, *RCS* retrospective controlled studies, *NS* not stated, *SA* spinal anesthesia, *GA* general anesthesia

### Quality assessment of the included studies

**Fig. 2:**
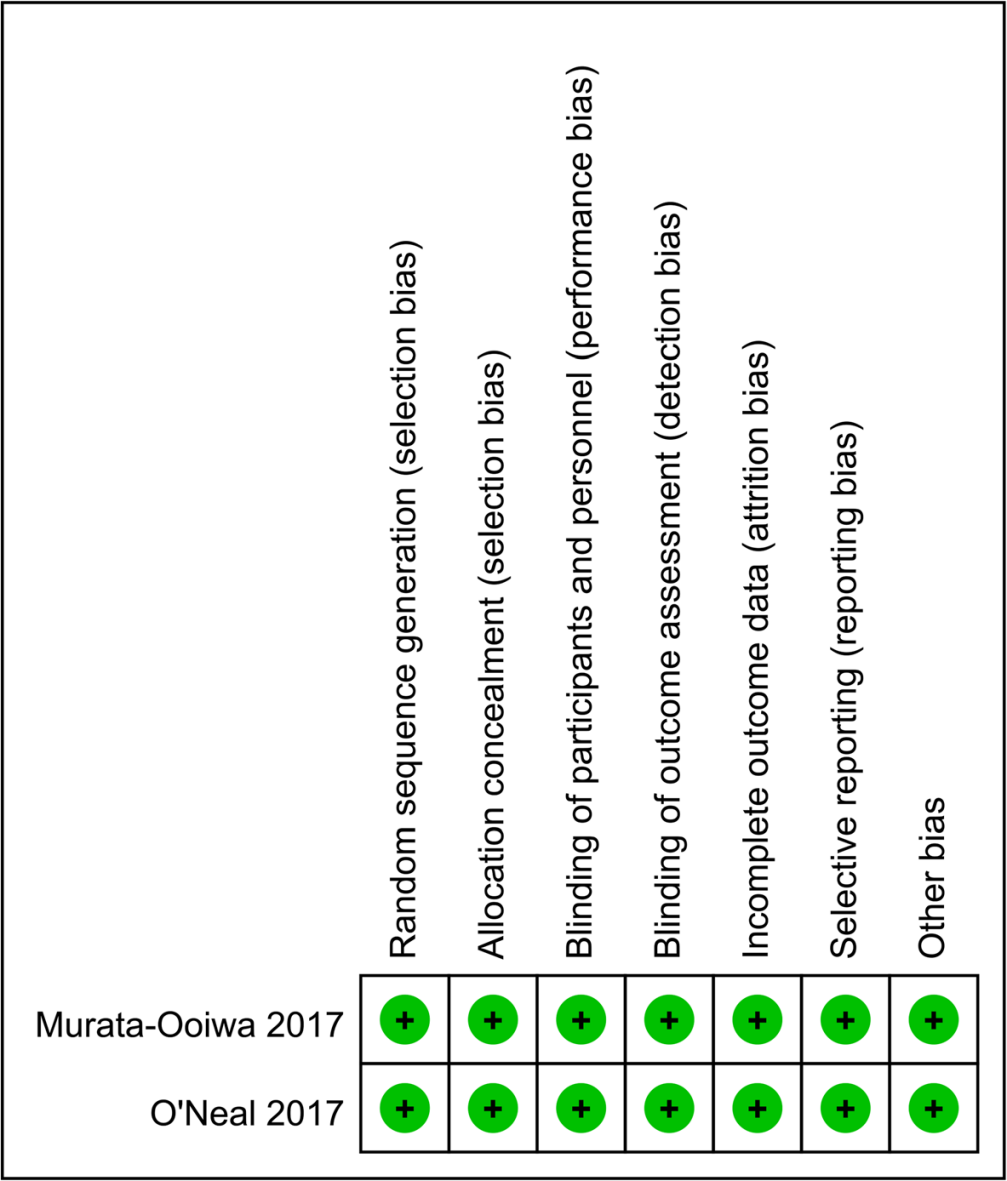
Risk of bias summary of the RCTs

**Fig. 3:**
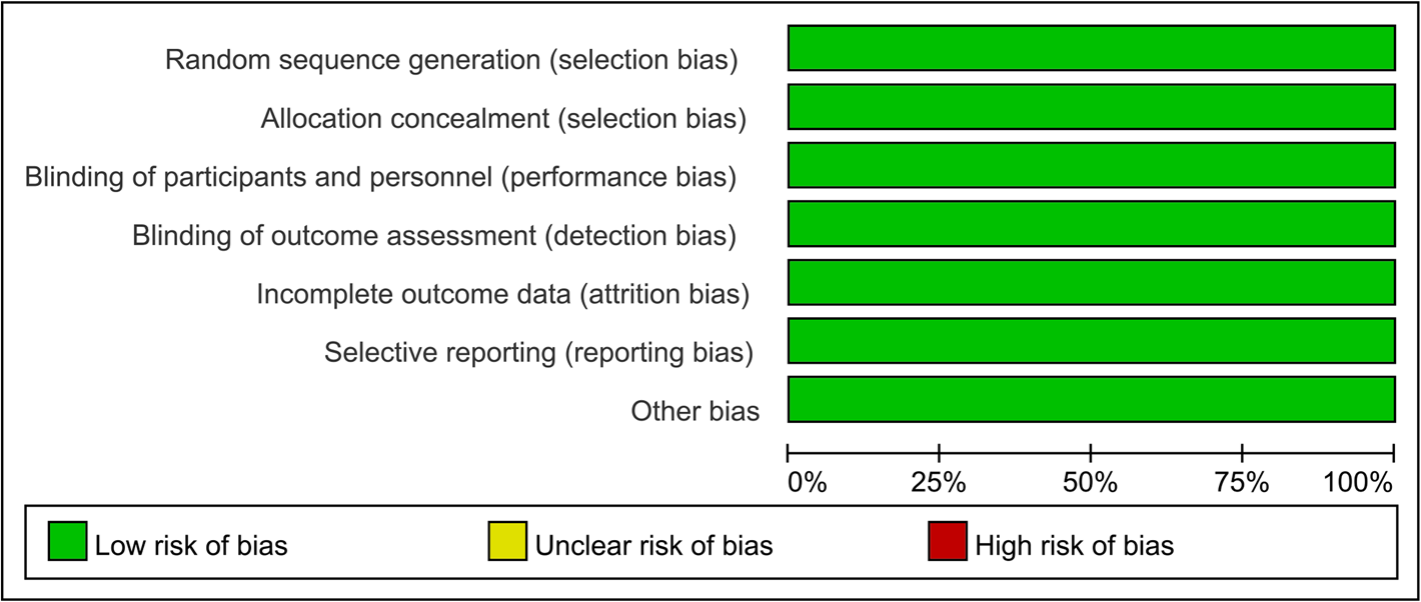
Risk of bias graph of the RCTs

The risk of bias summary and the risk of bias graph for the RCTs can be seen in Figs. 2 and 3, respectively. The two RCTs were both determined to be of high quality. The quality assessments of the non-RCTs can be seen in Table 2. Total scores of NOS ranged from 6 to 8.

**Table 2:** Newcastle-Ottawa scale for the non-RCTs

Author	Selection	Comparability	Outcomes	Total score
Kelly [15]	***	**	**	7
Nwagbologu [17]	**	**	**	6
Ciummo [23]	***	**	***	8
Huang [21]	**	**	**	6

* represent 1 score

### Meta-analysis results

#### Total morphine equivalent consumption

**Fig. 4:**
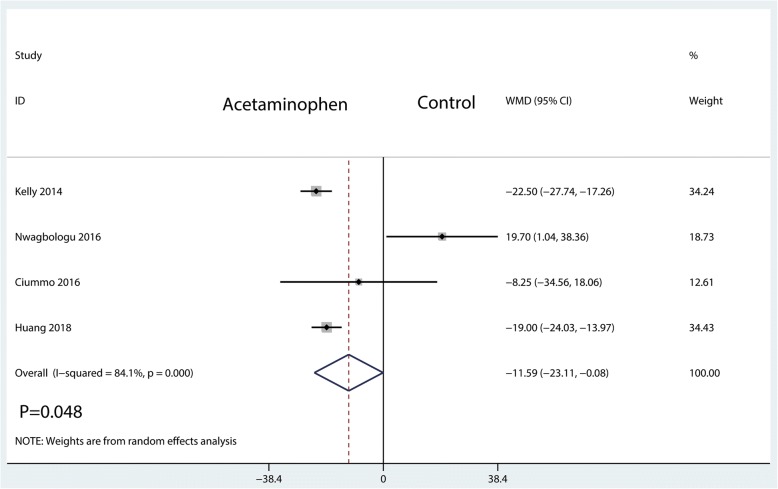
Forest plots of the included studies comparing the total morphine equivalent consumption

Four studies, having a total of 398 patients, reported equivalent total morphine consumptions. Compared with the control group, the IV acetaminophen group was associated with a reduction in total morphine consumption of approximately 11.59 mg (WMD = − 11.59; 95%CI, [− 23.11, − 0.08]; *P* = 0.048; *I*^2^ = 84.1%, Fig. 4).

#### Morphine equivalent consumption at POD 1

**Fig. 5:**
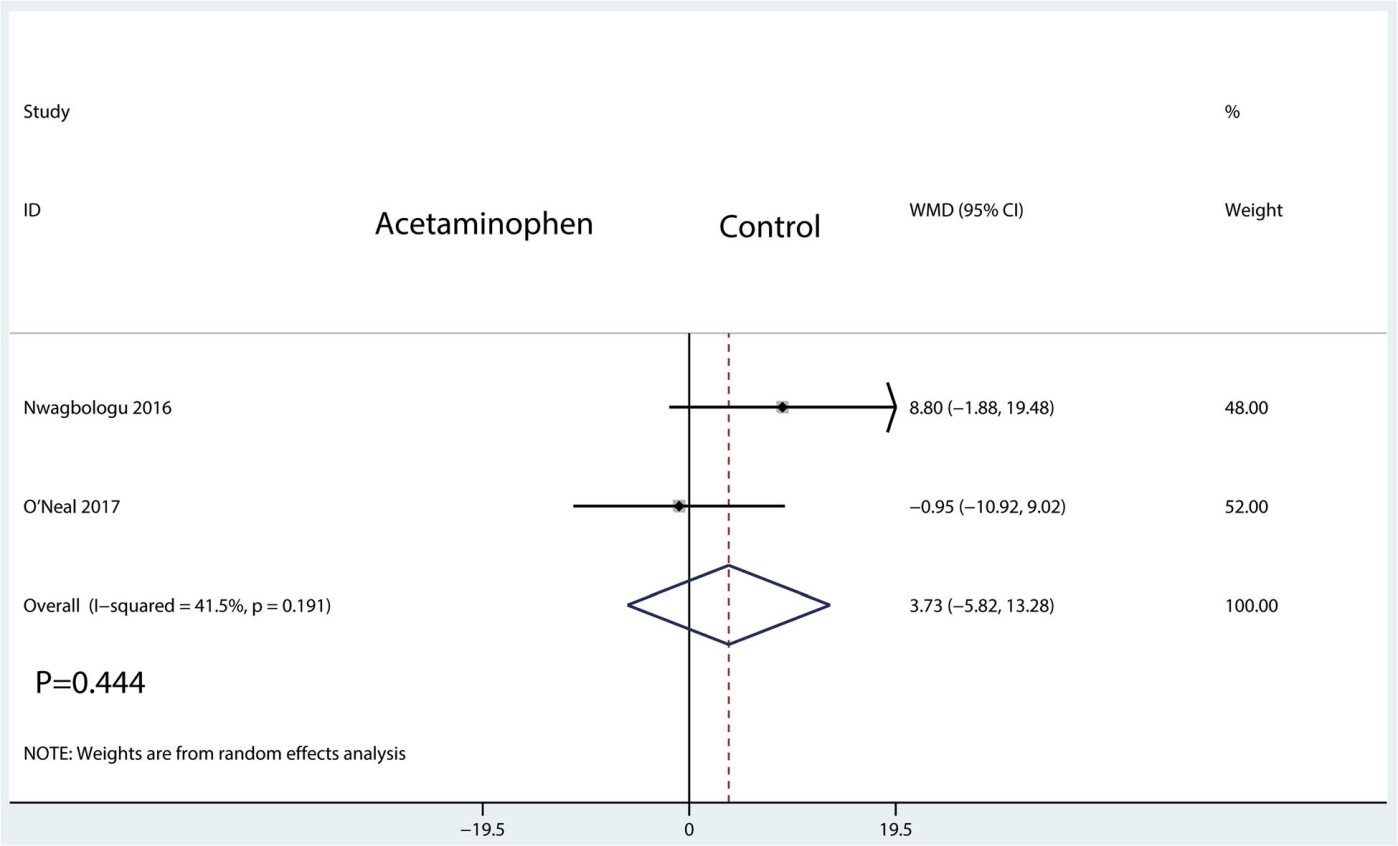
Forest plots of the included studies comparing the morphine equivalent consumption at POD 1

Data from 2 studies, including 264 patients, reported equivalent morphine consumption at POD 1. There were no significant differences between the IV acetaminophen group and control group in terms of morphine consumption at POD 1 (WMD = 3.73; 95%CI, [− 5.82, 13.28]; *P* = 0.444; *I*^2^ = 41.5%, Fig. 5).

#### Visual analogue scale at POD 1

**Fig. 6:**
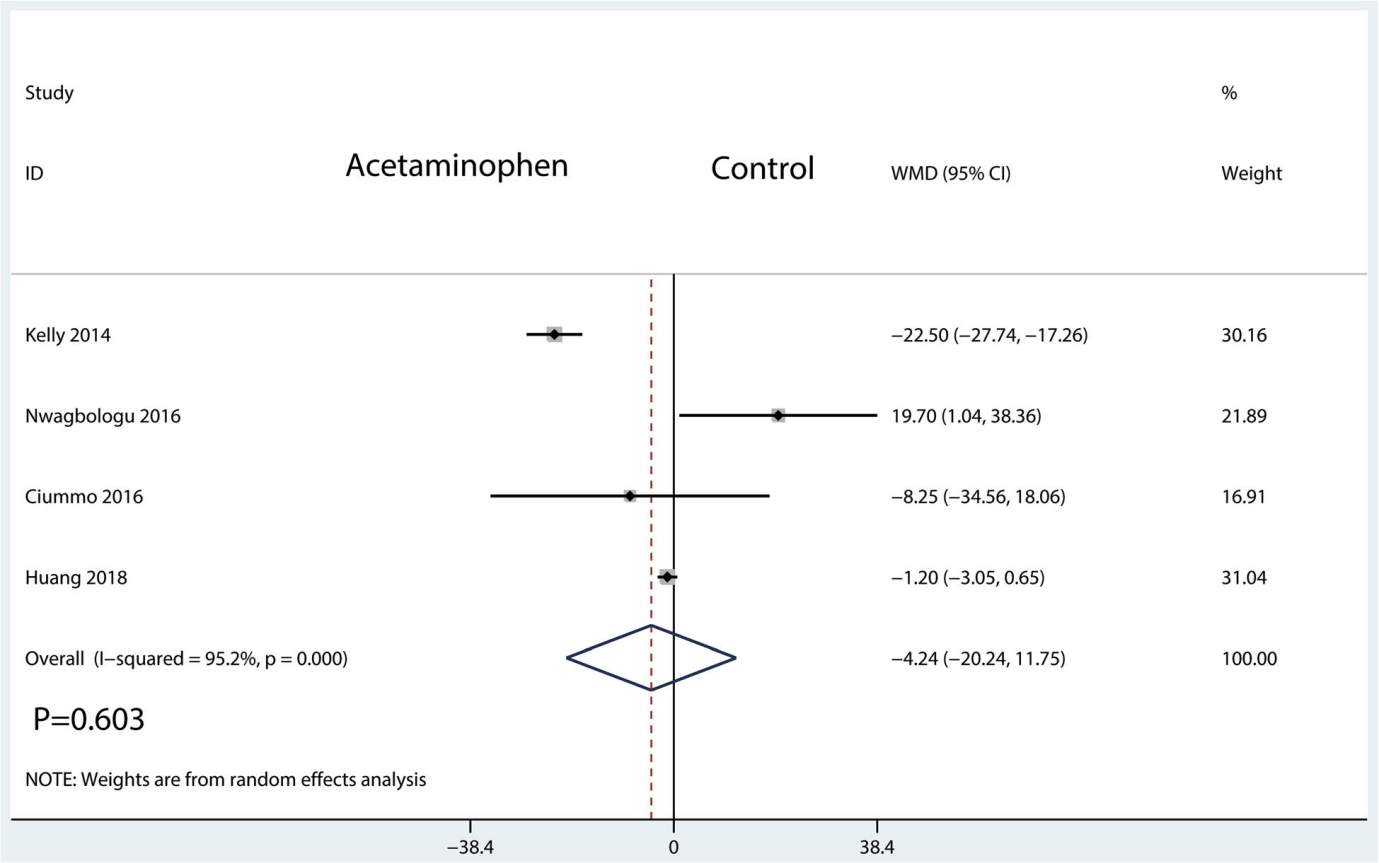
Forest plots of the included studies comparing the visual analogue scale score at POD 1

The visual analogue scale score at POD 1 was measured in 4 studies, having a total of 216 patients. We did not find any significant difference between the IV acetaminophen and control groups (WMD = − 4.24; 95%CI, [− 20.24, 11.75]; *P* = 0.603; *I*^2^ = 95.2%, Fig. 6).

#### Visual analogue scale at POD 2

**Fig. 7:**
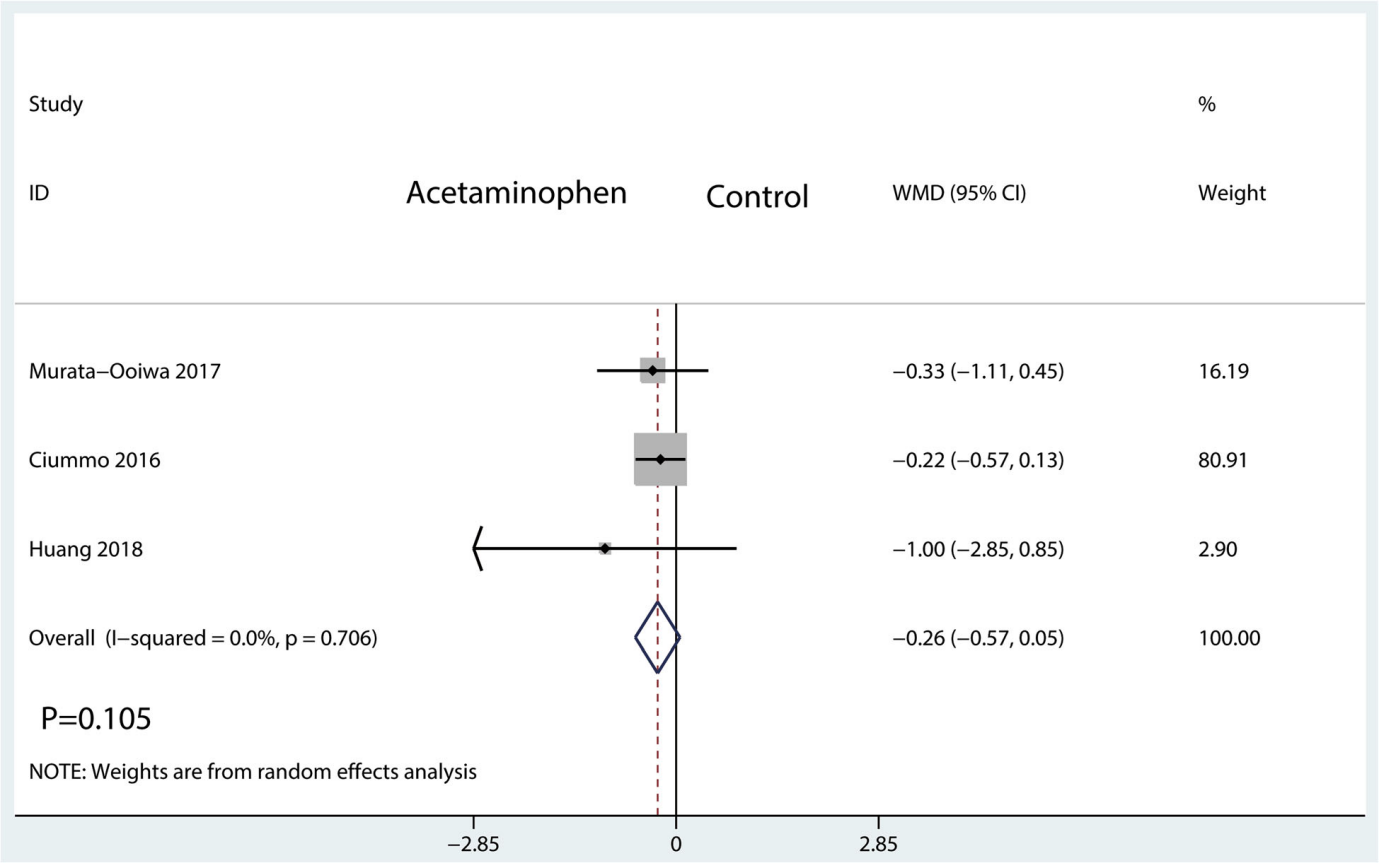
Forest plots of the included studies comparing the visual analogue scale score at POD 2

The visual analogue scale score at POD 2 was measured in 3 studies, having a total of 216 patients. There was no significant difference between IV acetaminophen and control groups in terms of the VAS score at POD 2 (WMD = − 0.26; 95%CI, [− 0.57, 0.05]; *P* = 0.105; *I*^2^ = 0.0%, Fig. 7).

#### Visual analogue scale at POD 3

**Fig. 8:**
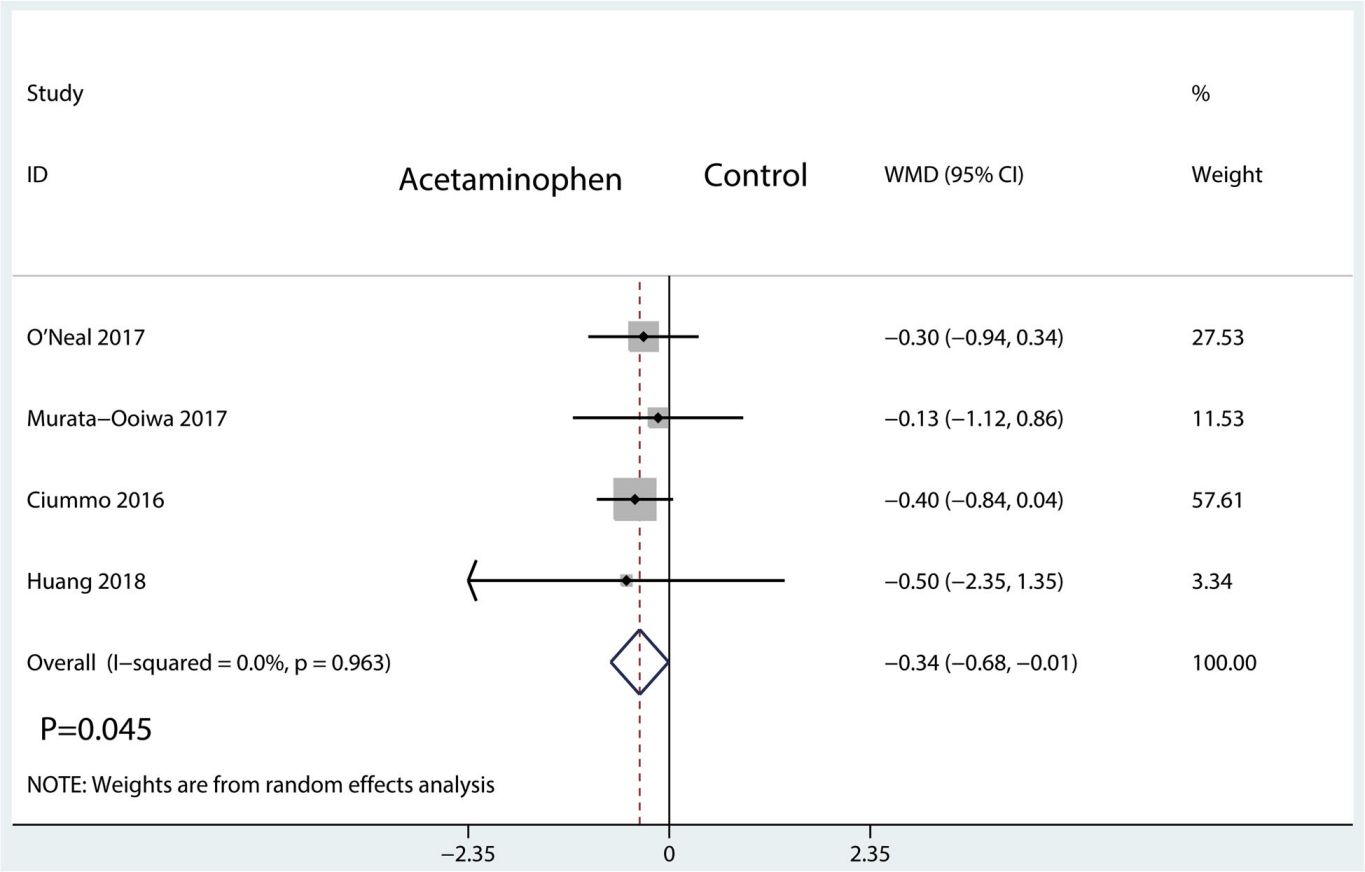
Forest plots of the included studies comparing the visual analogue scale score at POD 3

The visual analogue scale score at POD 3 was reported in 4 studies, having a total of 331 patients. Compared with the control group, the IV acetaminophen group was associated with a reduction in VAS score at POD 3 (WMD = − 0.34; 95%CI, [− 0.68, − 0.01]; *P* = 0.045; *I*^2^ = 0.0%, Fig. 8).

#### Length of hospital stay

**Fig. 9:**
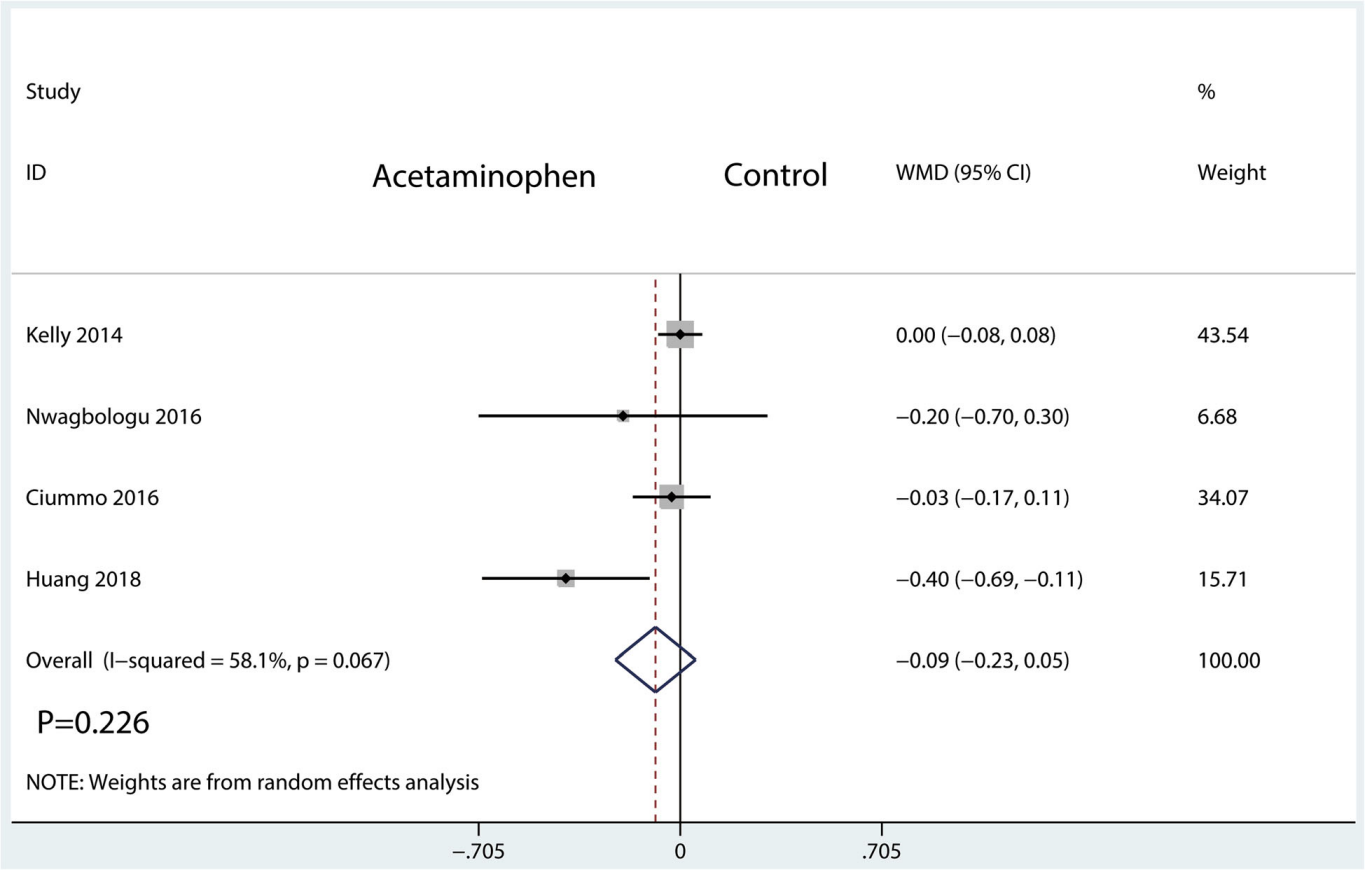
Forest plots of the included studies comparing the length of hospital stay

We extracted length of hospital stay data from 4 studies, involving 398 patients. There were no significant differences between the IV acetaminophen and control groups in terms of the length of hospital stay (WMD = − 0.09; 95%CI, [− 0.23, 0.05]; *P* = 0.226; *I*^2^ = 58.1%, Fig. 9).

#### Sensitivity analysis and subgroup analysis

The sensitivity analysis can be seen in Additional file 1**:** Figure S1. The results showed that after omitting each study in turn, the overall effects were in the upper CI limit and lower CI limit.

Subgroup analysis results can be seen in Table 3. The findings of decreased total morphine consumption were consistent in all subgroup analyses except for the allocation concealment and anesthesia subgroups.

**Table 3:** Subgroup analysis of the total morphine consumption

Subgroups	No. of studies	Mean difference [95% CI]	*P* value	*I*^2^ (%)	Between subgroup significance
Total morphine consumption					
Allocation concealment					
Adequate	2	**−** 2.39 (− 43.70, 38.92)	0.910	94.5	0.002
Unclear	2	− 18.62 (− 23.56, − 0.08)	0.000	0	
Study type					
RCT	1	− 19.70 (− 22.35, − 15.43)	0.029	0	0.124
RCS	3	− 20.45 (− 24.04, − 16.85)	0.038	0	
Acetaminophen dose					
1000 mg/day	3	− 4.42 (− 33.04, 24.20)	0.762	89.4	0.139
4000 mg/day	1	− 19.00 (− 24.03, − 13.97)	0.000		
Anesthesia					
GA	2	− 2.39 (− 43.70, 38.92)	0.910	94.5	0.048
SA	2	− 18.62 (− 23.56, − 13.68)	0.000		

*GA* general anesthesia, *SA* spinal anesthesia, *RCT* randomized controlled trials, *RCS* retrospective controlled studies

## Discussion

The current meta-analysis indicated that compared with a control group, intravenous acetaminophen was associated with reductions in total morphine consumption and VAS score at POD 3. There was no significant difference in morphine consumption at POD 1 or in VAS score at POD 1 or 2. Moreover, there was no significant difference in length of hospital stay between the intravenous acetaminophen group and the control group.

Inadequate pain management following TKA may influence the functional recovery, increase opioid consumption, and contribute to several complications [24]. Recently, multimodal pain management has been widely applied in TKA [25]. Multimodal pain management usually includes two or more medications with different mechanisms, such as opioids, nonsteroidal anti-inflammatory medications, steroids, and epinephrine. It is worth noting that the usage of opioids is frequently associated with side effects, such as nausea, vomiting, and pruritus [26]. Moreover, it has been reported that the pain score became worse at 24 h after TKA. The rebounding pain of multimodal pain management after POD 1 remains an important issue in patients who have received TKA [9]. More recently, multimodal pain management with IV acetaminophen for postoperative pain management has generated much discussion [27].

It was reported that opioid consumption was reduced from 29 to 39% in patients who received IV acetaminophen compared to a placebo in orthopedic procedures [28]. IV acetaminophen has been shown to have efficacy for reducing the consumption of opioids [15]. Murata-Ooiwa et al. [19] demonstrated that the VAS score was significantly better in the intravenous acetaminophen group than the placebo group at day 1 after TKA, with no significant differences in terms of the rate of complications between the groups. They drew the conclusion that intravenous acetaminophen provided better pain relief for patients undergoing unilateral TKA.

However, recently, other studies have reported different conclusions [18, 23]. In the O’Neal et al. study [18], the VAS scores of IV acetaminophen and placebo groups were compared, as well as total the morphine consumption, among other parameters. No significant differences were found between all groups for any outcome. Nwagbologu et al. [17] reported that the use of IV acetaminophen was not associated with a decrease in opioid use, opioid-related side effects, or any other outcomes in patients who received TKA. The current meta-analysis indicated that IV acetaminophen was associated with a statistically significant reduction in total morphine consumption by approximately 11.59 mg compared with a control group.

There was significantly heterogeneity between the included studies (*I*^2^ = 84.1%). Despite performing sensitivity analysis to diminish the impact of heterogeneity, the effect of heterogeneity still could not be eliminated completely. In the sensitivity analysis, we found that the study of Nwagbologu et al. [17] was the source of this heterogeneity. Nwagbologu et al. [17] recorded that the IV acetaminophen group and placebo group received similar doses of total morphine equivalents at 24 and 48 h postoperatively. We analyze the reason as follows: (1) this was a retrospective cohort study and may have had a selection bias; (2) this study comprised two doses of acetaminophen (1000 mg/day and 2000 mg/day), so opioid-sparing effects may be related to the amount of IV acetaminophen received; (3) this study simply added onto other pain medication orders without any organized or concerted effort to use a multimodal pain regimen to reduce opioid consumption.

VAS score was also an important result in our meta-analysis. Current meta-analysis indicated that IV acetaminophen only has a beneficial role in reducing VAS score at POD 3. Murata-Ooiwa et al... [19] reported that the VAS score at 17:00 1 day after TKA was significantly better in the intravenous acetaminophen group than the placebo group. In contrast, some published studies have recently reported that IV acetaminophen has no effects on pain relief [18]. O’Neal et al. [18] reported that no significant differences were found between the IV acetaminophen and placebo groups regarding the VAS score. Similarly, Ciummo et al. [23] declared that there was no statistically significant difference in average daily postoperative VAS score.

Similar findings were reported by Murata-Ooiwa et al. [19]. In Murata-Ooiwa et al.’s study [19], there were no significant differences in the rate of complications. With regard to LOS, Ciummo et al. [23] reported that no significant differences were found. Kelly et al. [15] reported that the median length of LOS for both the IV acetaminophen and placebo groups was 3 days. In the Nwagbologu et al. study [17], the LOS in IV acetaminophen and control groups were 3.7 days and 3.9 days, respectively. These results were consistent with our meta-analysis. Therefore, we concluded that IV acetaminophen was not associated with a reduction in the length of hospital stay in patients who received TKA.

Our meta-analysis has several limitations: (1) Only six studies were included in our meta-analysis. The statistical efficacy of our results would be more reliable if more studies had been included. (2) Only English publications were included in our meta-analysis; therefore, publication bias was unavoidable. (3) Outcomes such as range of motion of the knee and knee society score were not analyzed due to insufficient data. (4) Follow-ups of these studies were relatively short, and long-term follow-ups are needed to identify the knee function between these two groups. (5) There was substantial heterogeneity between included outcomes. We performed subgroup analysis and sensitivity analysis to decrease the heterogeneity; however, the overall heterogeneity was not changed after subgroup analysis or after sensitivity analysis. Thus, the results of this meta-analysis should be carefully interpreted.

## Conclusion

In conclusion, based on our results, IV acetaminophen in multimodal management has shown better efficacy than a control for pain relief at POD 3 and has morphine-sparing effects. We identified six studies; in the future, the multimodal pain management protocol after TKA may change when more studies are published and included in the meta-analysis. Due to the limited studies and participants, further high-quality studies with more patients are needed to validate the optimal dose of IV-acetaminophen.

## Additional file

Additional file 1: **Figure S1.** Sensitivity analysis of the total morphine equivalent consumption (A), morphine equivalent consumption at POD 1 (B), visual analogue scale score at POD 1 (C), visual analogue scale score at POD 2 (D), visual analogue scale score at POD 3 (E), and length of hospital stay (F). (TIF 860 kb)

## Abbreviations

CI: Confidence interval
IV: Intravenous
NOS: Newcastle-Ottawa Quality Assessment Scale
OA: Osteoarthritis
POD: Postoperative day
RA: Rheumatoid arthritis
RCS: Retrospective cohort studies
RCTs: Randomized controlled trials
RR: Risk ratio
TKA: Total knee arthroplasty
VAS: Visual analogue scale
WMD: Weighted mean difference
