# Supplementary figures and images for: Efficacy of intravenous acetaminophen in multimodal management for pain relief following total knee arthroplasty: a meta-analysis
Source: J Orthop Surg Res. 2018 Oct 11;13:250. doi: 10.1186/s13018-018-0950-7 (PMC6180443; doi:10.1186/s13018-018-0950-7)

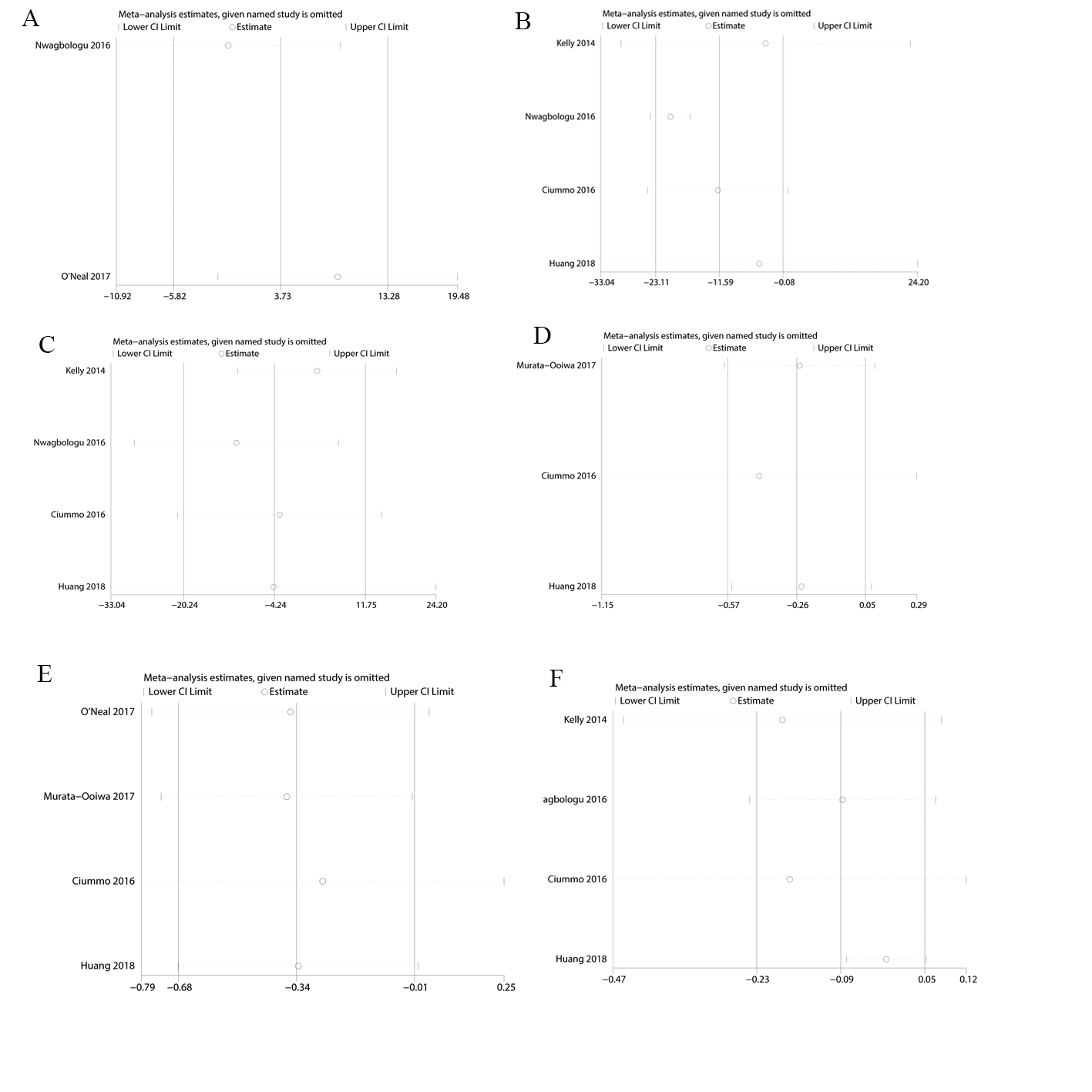

Supplement: Supplementary file 1 — Figure S1. Sensitivity analysis of the total morphine equivalent consumption (A), morphine equivalent consumption at POD 1 (B), visual analogue scale score at POD 1 (C), visual analogue scale score at POD 2 (D), visual analogue scale score at POD 3 (E), and length of hospital stay (F). (TIF 860 kb) [file 13018_2018_950_MOESM1_ESM.tif]
